# Beyond the Bite: Exploring Mental Health of Dental Faculty in Pakistan– a multi-institutional study

**DOI:** 10.1038/s41405-024-00263-y

**Published:** 2024-10-18

**Authors:** Kamran Ali, Daniel Zahra, Ulfat Bashir, Alaa Daud, Hina Zafar Raja, Rob Witton, Mahwish Raja

**Affiliations:** 1 Qatar University, QU Health College of Dental Medicine, Doha, 2713 Qatar; 2 Plymouth University, School of Psychology, Plymouth, PL4 8AA UK; 3 Islamic International Dental College Riphah International University, Islamabad, Pakistan; 4 CMH Institute of Dentistry, Lahore, Pakistan; 5 Plymouth University, Peninsula Dental School, Plymouth, PL4 8AA UK

**Keywords:** Health care, Dental psychology

## Abstract

**Introduction:**

Mental health issues are being reported increasingly amongst healthcare staff and students globally. The aim of this study was to investigate the frequency of common mental health issues amongst dental faculty members at multiple institutions in Pakistan.

**Methods:**

Following approval from the institutional ethics review board, dental faculty members at 14 dental institutions were invited to participate in an online survey based on globally validated scales for mental health problems including the Patient Health Questionnaire (PHQ-9), and the Depression, Anxiety, and Stress Scale (DASS-21). Two open-ended questions were included in the survey to identify perceived factors contributing to poor mental health and recommendations for improving institutional support.

**Results:**

A total of 200 faculty members out of provided their responses to the survey questionnaire but complete responses were provided by 183 participants which included 120 (65.57%) females, and 63 (34.43%) males. The total number of faculty members at the participating institutions was 426 and 183 responses translated into an overall response rate of 43%. Most participants were in the 31–40 years age-group (*n* = 81, 44.26%) followed by 25–30 year (*n* = 51, 22.87%) and 41–50 years (*n* = 40, 21.86%). The mean score on PHQ-9 was 6.51 (SD ± 5.4) while the mean DASS-21 score was 13.04 (SD ± 10.95). PHQ-9 Depression, and DASS-21 Depression, Anxiety, and Stress scores were all significantly positively correlated for the whole sample, and within each subgroup of each demographic factor. Job-related workload, lack of institutional support, financial limitations, and poor work life balance were identified as the main factors contributing adversely to the mental health of the participants.

**Discussion:**

This study provides useful insights into the scale of mental health status amongst dental faculty members at 14 institutions in Pakistan. Underlying factors affecting the mental health of faculty members adversely were identified and recommendations are provided to address these challenges.

## Introduction

Mental health is a state of well-being in which individuals realize their own potential, cope with normal stresses of life, work productively, and contribute to their community, and is a critical component of overall health [1]. Despite its importance to overall health, mental health issues are prevalent worldwide, impacting individuals across various walks of life. According to the WHO, one in four people in the world will be affected by mental or neurological disorders at some point in their lives, placing mental disorders among the leading causes of ill-health and disability globally [2]. Mental health issues encompass a wide range of conditions, from common disorders like depression and anxiety to more severe conditions such as schizophrenia and bipolar disorder. The impact of these disorders is profound; the Global Burden of Disease Study reported that mental health disorders are major contributors to the overall global burden of disease [3]. Importantly, mental health is influenced by a multitude of factors, including biological, environmental, and social elements, underlining the need for comprehensive approaches in both understanding and addressing mental health issues [4]. The ongoing challenge in mental health care is not only treating the conditions but also destigmatizing mental health issues, promoting mental well-being, and implementing effective prevention strategies.

Mental health issues within academia and among faculty members are increasingly recognized as significant concerns impacting the well-being of educators and the quality of education they provide. The academic environment, known for its high-pressure demands such as “publish-or-perish” culture, grant acquisition stress, and the juggling of teaching, research, and administrative responsibilities, creates a unique set of challenges for mental health. A study by Kinman and Wray (2021) highlights that academic staff experience higher levels of work-related stress, anxiety, and depression compared to other professional sectors [5]. This is compounded by the precarious nature of academic employment and the intensity of academic workloads [6]. These stressors can lead to burnout, a state of emotional, mental, and often physical exhaustion caused by prolonged stress, as identified in Maslach et al.‘s (2001) seminal work on the subject. The impact of these mental health issues is not limited to the individuals suffering from them but extends to the educational environment, potentially affecting teaching quality, student support, and overall academic productivity [7]. As such, addressing mental health in academia is not just a matter of individual well-being, but a strategic imperative for institutions aiming to foster a healthy, effective educational setting.

Mental health among dental faculty has emerged as a critical concern, warranting increased attention due to its significant impact on educators, students, and the overall educational environment. In dental academia, these pressures are often compounded by the demands of clinical duties, research responsibilities, and the need to stay abreast of rapidly evolving technologies and treatment modalities.

The consequences of unaddressed mental health issues in this sector are far-reaching. They not only affect the well-being and productivity of the faculty but also impact the quality of education and mentorship they provide to their students. Faculty burnout can lead to reduced academic productivity and diminished quality of teaching, which in turn can influence the learning environment and outcomes for students [8]. Furthermore, there is a risk of perpetuating a cycle where stressed and burned-out educators model unhealthy work habits to their students, who may carry these patterns into their professional lives [9].

The aim of this study was to evaluate the mental health of dental faculty at institutions in Pakistan.

## Methods

### Research ethics

Ethical approval was obtained from the ethical review board (ANMC/ERB/23 dated 16/02/2023). Participation was voluntary and all data were collected and processed anonymously. All participating faculty members provided consent before providing responses to the study questionnaire.

### Study design

An analytical cross-sectional design based on an online survey using google forms was employed for this study.

### Sample size calculation

The sample size for this study was determined using a power analysis with G*Power software (version 3.1) [10]. In the Chi-squared analyses reported, where the degrees of freedom are between 8 and 12, using α = 0.05, the total sample size required to maintain a power of 0.90, and detect small-to-medium effects (w = 0.2) is between *n* = 175 and *n* = 207. These are also sufficient to detect small effect sizes using analyses such as t-test to compare differences in mean scores between independent groups.

### Sampling technique and participants

A probability sampling technique was used to target dental faculty involved in teaching dental students at 14 dental institutions. Faculty who had interrupted work were excluded.

The target participants were approached through their Head of the institution and invited to participate in an online survey. The office of the Head of the institution acted as the gatekeeper for sending the invites. A reminder was sent two weeks later.

### Data collection instruments

The survey questionnaire consisted of five structured sections and was administered online using google forms.

The initial section pertained to obtaining informed consent from participants, confirming their voluntary participation and comprehension of the study’s purpose and scope, with assurance of anonymized data processing. The subsequent section captured demographic data, encompassing age, gender, institution, and details of scope of work (specialty) of the participants. The third segment utilized a nine-item version of the Patient Health Questionnaire (PHQ-9), a validated tool widely employed for depression screening. Following this, the fourth section employed the 21-item Depression, Anxiety, and Stress Scale (DASS21), also validated for mental health screening (Jiyeon et al., 2019). Each of the DASS21 subscales comprised seven items scored on a Likert scale from 0 to 3. The final section encompassed two open-ended inquiries concerning factors influencing participants’ mental health and recommendations for enhancing support for faculty facing mental health challenges.

### Data analysis

Analysis of the data was performed utilizing the R statistical environment for Windows (R Core Team, 2022) [11]. Descriptive statistics were computed to delineate the sample and subgroups, as well as to depict the distribution of scores across each scale and among various levels of each factor. Chi-squared tests of association were employed to ascertain differences in severity category distribution across groups for each scale. Pearson’s correlation coefficients were utilized to assess the relationships among scale scores.

## Results

### Demographics

Of the 200 responses received, 183 provided complete PHQ-9 and DASS-21 scales, and these form the basis of the analyses; participants with any missing PHQ-9 or DASS-21 item responses were excluded as thresholds are based on summation across items and scaling scores to account for missing data would complicate interpretation of the findings. The total number of faculty members at the participating institutions was 426 and 183 responses translated into an overall response rate of 43%. Respondent characteristics for these 183 complete responses are summarised in Table 1.

**Table 1:** Summary of respondent characteristics.

Factor	Level	*n*	Total (Percentage)
Gender	Female	120	65.57
	Male	63	34.43
Age Group	25–30	51	27.87
	31–40	81	44.26
	41–50	40	21.86
	51–60	8	4.37
	Over 60	3	1.64
Institution Type	Public	28	15.30
	Private	151	82.51
	Other	4	2.19
Region	Baluchistan	18	9.84
	Islamabad	53	28.96
	KPK	17	9.29
	Punjab	88	48.09
	Sindh	7	3.83
Years Experience	<5	78	42.62
	5–10	55	30.05
	11–15	21	11.48
	16–20	17	9.29
	21–25	9	4.92
	>25	3	1.64
Specialty	Basic Sciences	59	32.24
	Clinical	115	62.84
	Clinical and/or Med. Ed.	9	4.92

### Descriptive statistics

PHQ-9 responses to each item were scored as follows: Not at all = 0, Several days = 1, More than half the days = 2, and Nearly every day = 3. Item scores were summed across the nine items to provide an overall PHQ-9 score, which was then categorised into the following depression severity groups: None (0–4), Mild (5–9), Moderate (10–14), Moderately Severe (15–19), and Severe (20–27).

DASS-21 responses to each item were scored as follows: Did not apply to me at all = 0, Applied to me to some degree, or some of the time = 1, Applied to me to a considerable degree or a good part of time = 2, Applied to me very much or most of the time = 3. Items scores were then summed to give an overall DASS-21 score, and groups of items summed to provide Depression (Items 3, 5, 10, 13, 16, 17, 21), Anxiety (Items 2, 4, 7, 9, 15, 19, 20), and Stress (Items 1, 6, 8, 11, 12, 14, 18) subscale scores. These subscale scores were then used to categorise the severity of each dimension using thresholds shown in Table 2.

**Table 2:** DASS-21 subscale severity category thresholds.

Severity Category	DASS-21 Subscale		
	Depression	Anxiety	Stress
Normal	0–4	0–3	0–7
Mild	5–6	4–5	8–9
Moderate	7–10	6–7	10–12
Severe	11–13	8–9	13–16
Extremely Severe	14+	10+	17+

Descriptive statistics for each scale score and the numbers of respondents in each category are shown in Tables 3 and 4.

**Table 3:** PHQ-9 and DASS-21 descriptive statistics by scale and subscale.

Statistic	PHQ-9	DASS-21			
	Total	Total	Depression	Anxiety	Stress
Mean	6.51	13.04	4.25	3.26	5.52
SD	5.44	10.95	4.32	3.29	4.20
Min	0	0	0	0	0
Max	25	55	19	15	21
Range	25	55	19	15	21
IQR	7	15	5.5	5	5

**Table 4:** Categorisation by scale and subscale.

Category	Frequency				Percentage of Respondents			
	PHQ9	DASS-21			PHQ9	DASS-21		
		Depression	Anxiety	Stress		Depression	Anxiety	Stress
None	78	–	–	–	42.62	–	–	–
Normal	–	113	112	138	–	61.75	61.20	75.41
Mild	60	24	27	14	32.79	13.11	14.75	7.65
Moderate	27	25	21	18	14.75	13.66	11.48	9.84
Moderately Severe	13	–	–	–	7.10	–	–	–
Severe	5	16	13	9	2.73	8.74	7.10	4.92
Extremely Severe	–	5	10	4	–	2.73	5.46	2.19

PHQ-9, DASS-21 Depression, Anxiety, and Stress scores were all significantly positively correlated for the whole sample, and within the vast majority of subgroups of each factor. Whole-sample correlation coefficients are shown in Table 5.

**Table 5:** Pearson Correlation Coefficients (r) for correlations between each scale. Values based on all complete responses. All values are statistically significant at *p* < 0.001.

		DASS-21		
		Depression	Anxiety	Stress
**PHQ-9 Total**		0.76	0.58	0.70
**DASS21**	**Depression**	–	0.75	0.84
	**Anxiety**	–	–	0.75

#### PHQ-9

##### Gender

**Fig. 1:**
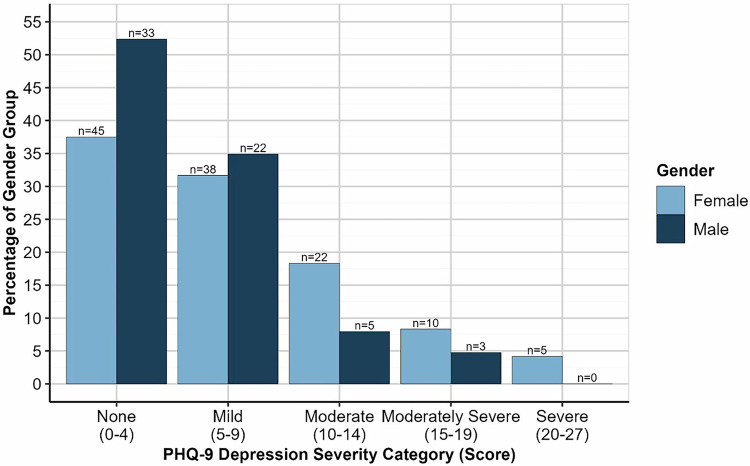
PHQ-9 depression severity category by gender. Patient Health Questionnaire-9.

Female respondents scored significantly higher on the PHQ-9 (*M* = 7.31, SD = 5.72) than Male respondents (*M* = 4.98, SD = 4.54; *t*(152.95) = 3.00, *p* = 0.003). However, the distribution of depression severity categories between genders were comparable; *Χ*^2^(4) = 8.67, *p* = 0.07 (Fig. 1).

##### Years of experience

Years of Experience was found to have an overall main effect on PHQ-9 scores; *F*(5,177) = 2.38, *p* = 0.040, although these differences are not reflected in associations between Years of Experience and PHQ-9 depression severity category, *Χ*^2^(20) = 18.17, *p* = 0.577. This also holds when omitting the smaller 21–25 and >25 years’ experience groups, *Χ*^2^(12) = 10.85, *p* = 0.542. Variation in PHQ9 scores is summarised in Table 6.

**Table 6:** PHQ-9 depression severity category by years of experience.

Years		PHQ-9 Depression Severity Category					
		None	Mild	Moderate	Moderately Severe	Severe	Total
<5	*n*	26	30	12	8	2	78
	%	33.33	38.46	15.38	10.26	2.56	100
5–10	*n*	25	16	6	5	3	55
	%	45.45	29.09	10.91	9.09	5.45	100
11–15	*n*	9	7	5	0	0	21
	%	42.86	33.33	23.81	0.00	0.00	100
16–20	*n*	9	5	3	0	0	17
	%	52.94	29.41	17.65	0.00	0.00	100
21–25	*n*	6	2	1	0	0	9
	%	66.67	22.22	11.11	0.00	0.00	100
>25	*n*	3	0	0	0	0	3
	%	100.00	0.00	0.00	0.00	0.00	100

##### Specialty

Specialty (Basic Sciences vs. Clinical; Clinical and Medical Education at *n* = 9 was excluded) showed no association with PHQ-9 depression severity category; *Χ*^2^(4) = 4.55, *p* = 0.337.

##### Institution type

Institution Type (Public vs. Private; Other at *n* = 4 was excluded) showed no association with PHQ-9 depression severity category; *Χ*^2^(4) = 4.44, *p* = 0.350.

##### Region

Region (excluding Sindh, *n* = 7) showed no association with PHQ-9 depression severity category; *Χ*^2^(16) = 15.88, *p* = 0.462.

##### Age -group

Age-group (omitting those over 50) showed no association with PHQ-9 depression severity category; Χ^2^(8) = 13.20, *p* = 0.105. Participants in the 51–60 and over 60-year age-groups were less than 5% of the sample and were too small to provide meaningful statistical analysis. Therefore, they were be excluded to avoid unreliable conclusions.

#### DASS-21

##### Gender

**Fig. 2:**
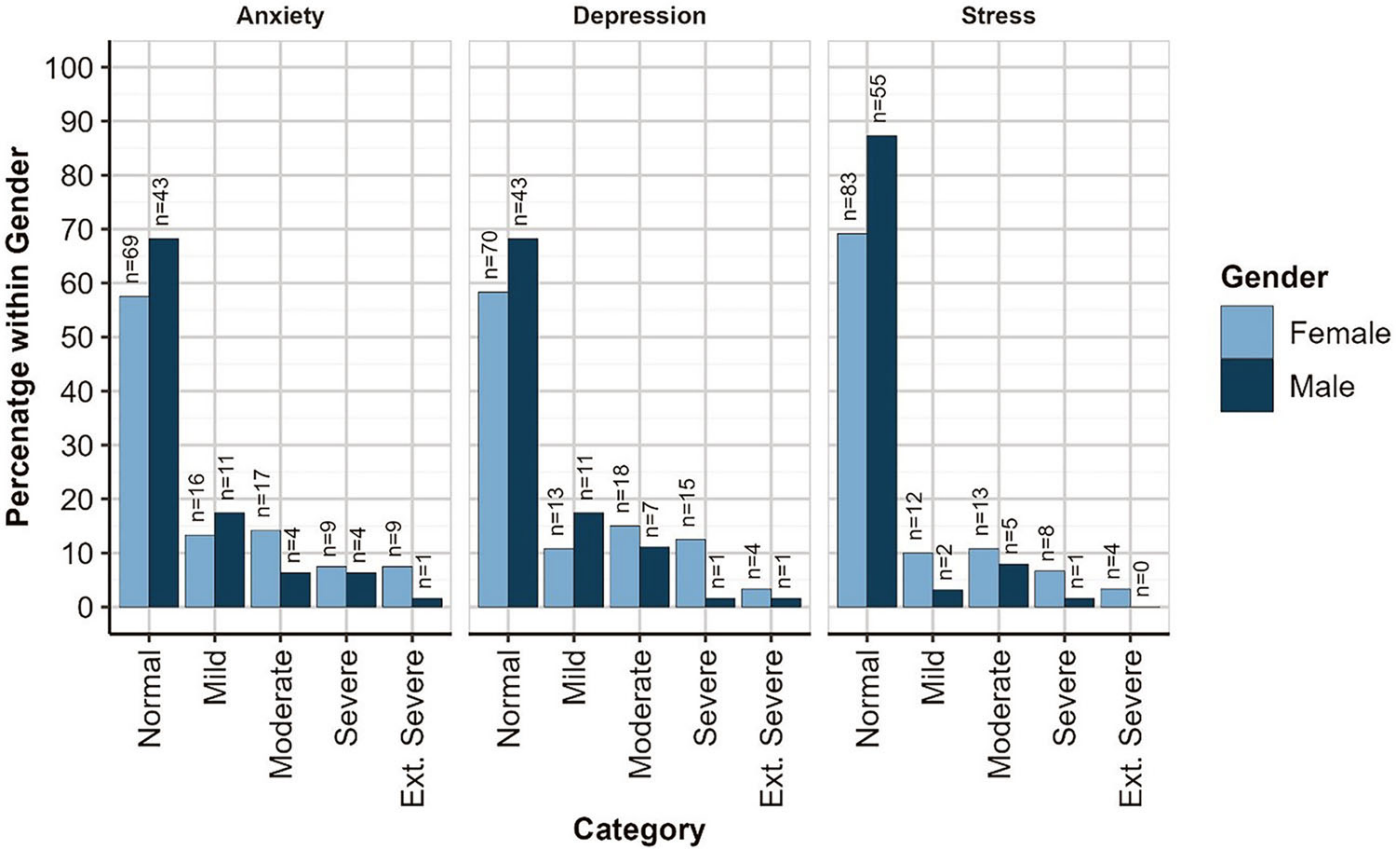
DASS-21 subscale severity category by gender. Depression, Anxiety, Stress Scale -21.

Comparable proportions of Males and Females are found in each DASS-21 Depression (*Χ*^2^(4) = 8.59, *p* = 0.072), Anxiety (*Χ*^2^(4) = 6.18, *p* = 0.186), and Stress (*Χ*^2^(4) = 8.94, *p* = 0.063) severity categories; distributions are shown in Fig. 2.

##### Years of experience

Years of Experience was not associated with DASS-21 Depression (*Χ*^2^(20) = 14.06, *p* = 0.827), Anxiety (*Χ*^2^(20) = 13.63, *p* = 0.849), or Stress severity category (*Χ*^2^(20) = 16.82, *p* = 0.665); this absence of associations also held when omitting the smaller 21–25 and >25 years’ experience groups.

##### Specialty

Specialty (Basic Sciences vs. Clinical; Clinical and Medical Education at *n* = 9 was excluded) showed no association with DASS-21 Depression (Χ^2^(4) = 3.92, *p* = 0.417), Anxiety (Χ^2^(4) = 4.61, *p* = 0.329), or Stress severity categories (Χ^2^(4) = 6.98, *p* = 0.137).

##### Institution type

Institution Type (Public vs. Private; Other at *n* = 4 was excluded) showed no association with DASS-21 Depression (Χ^2^(4) = 3.50, *p* = 0.478), Anxiety (Χ^2^(4) = 2.18, *p* = 0.702), or Stress severity categories (Χ^2^(4) = 4.33, *p* = 0.363).

##### Region

Region (excluding Sindh, *n* = 7) showed no association with DASS-21 Depression (Χ^2^(4) = 2.24, *p* = 0.691), Anxiety (Χ^2^(4) = 3.99, *p* = 0.407), or Stress severity categories (Χ^2^(4) = 3.01, *p* = 0.557).

##### Age -group

**Fig. 3:**
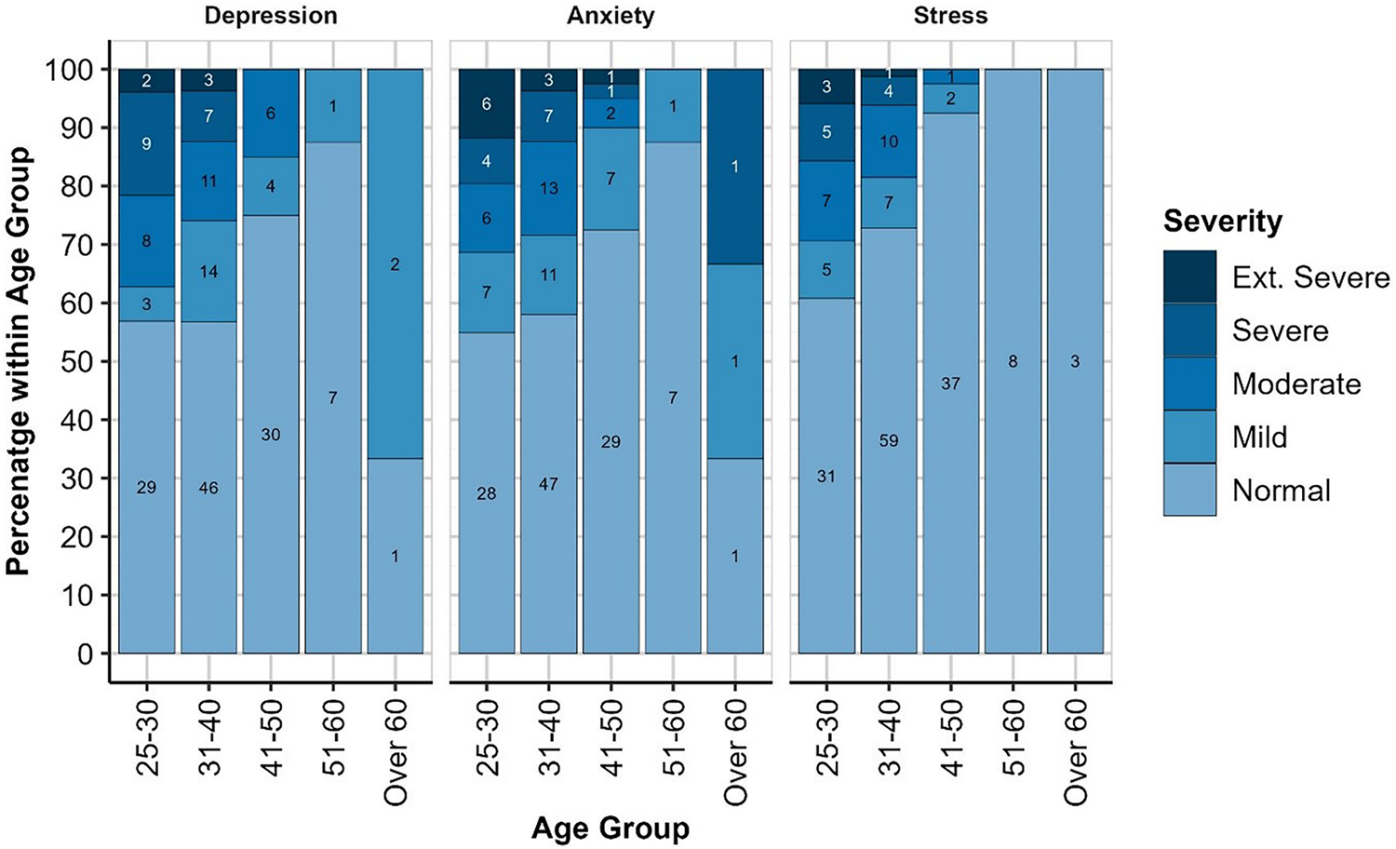
DASS-21 subscale severity by age group. Values within each bar indicate the frequency of respondents in that category.

Age Group was associated with DASS-21 Stress severity (*Χ*^2^(8) = 15.237, *p* = 0.050), but not Depression (*Χ*^2^(8) = 14.40, *p* = 0.067) or Anxiety severity (*Χ*2(8) = 10.31, *p* = 0.247). As with PHQ-9, participants in the 51–60 and over 60-year age-groups were less than 5% of the sample and were excluded. Severity category distributions for each subscale by age-group are shown in Fig. 3.

### Responses to open ended questions

Responses to open-ended questions were analysed thematically. The responses were organized and read systematically to identify recurring patterns of responses. Coding of relevant sections of the texts was done and then grouped into primary themes (sub-themes) and related elements were then combined into higher level themes.

The most common factor was workload related to job commitments and followed by institutional factors, economic conditions of the country and challenges of maintaining a work life balance. Underlying factors perceived to affect the mental health under each theme are summarised in Table 7.

**Table 7:** Underlying factors affecting mental health of participants.

Theme	Sub-themes	Frequency*
*I. Workload*	⚬ Stress associated with multitasking ⚬ Heavy workload ⚬ Unrealistic targets	++++
*II. Institutional factors*	⚬ Lack of recognition and reward ⚬ Favouritism and injustice ⚬ Bullying, injustice, favouritism, and judgemental behaviours by senior management ⚬ Delays in disbursement of salary ⚬ Poor facilities and infrastructure ⚬ Job insecurity	++++
*III. Economic conditions*	⚬ High inflation and living costs ⚬ Limited career opportunities in the current job market ⚬ Poor political and financial climate in the country	+++
*Poor work life balance*	⚬ Long working hours ⚬ Daily commute to workplace ⚬ Need to do additional work-related tasks at home after work	+++

Each + represents 10% participants.

The participants also provided several recommendations to improve the support for faculty to minimize adverse impact of work-related stress, anxiety and depression, as summarised in Table 8. The responses underscored the need to improve the working conditions for the faculty, enhancement of financial packages for the faculty and measures to improve institutional support for the faculty.

**Table 8:** Recommendations of the participants to improve mental health of students.

Theme	Sub-themes	Frequency*
*I. Improve working conditions*	• Reduce academic and clinical workload • Provide more administrative support staff • Provide protected time for research • Arrange training activities to enhance professional growth of faculty • Improve institutional infrastructure • Better opportunities for social and recreational activities for faculty	++++
*II. Enhancement of financial package*	• Need to increase salary to reflect market inflation • Offer financial bonuses based on performance • Provide better job contract to enhance job security	+++
*III. Institutional support*	• Flexibility in working hours for faculty with children and elderly family members requiring care • Fully paid maternity leave • Childcare facilities • Provide free access to mental health support services for staff experiencing poor mental health	++

Each + represents 10% participants.

## Discussion

Employment may be viewed as an opportunity which enhances an individual’s quality of life, playing a crucial role in everyday experiences. It significantly influences the development of an individual’s social identity and promotes social integration, owing to its economic and cultural dimensions. However, what is intended to be a means of financial autonomy and personal satisfaction can, under certain circumstances, transform into a source of psychological strain. This shift often occurs due to work-related fatigue and stress, potentially leading to various psychological disorders. Engagement with work is a positive, fulfilling state, yet when overextended, it can lead to burnout, characterized by exhaustion and a cynical attitude towards work [12]. Other studies have also reported the impact of prolonged job stress, leading to emotional exhaustion, depersonalization, and reduced personal accomplishment – key components of job burnout. These findings highlight the dual nature of work as both a source of personal fulfillment and, potentially, a trigger for mental health challenges [7].

This is a maiden study exploring the mental health of dental faculty from multiple institutions in Pakistan. Whilst there are numerous published studies investigating the mental health of dental and medical students, less is known how employment as a dental faculty affects the mental health of incumbents. Similarly, there is a plethora of published literature on the frequency of burn out and compassion fatigue in healthcare professionals globally, especially following the COVID-19 pandemic [13–17]. However, less is known about the frequency of stress, anxiety, depression and related mental health issues amongst dental academics. Dental education involves training novice dental students in a variety of settings including classrooms, simulation laboratories, and clinical environments. One of the most challenging aspects of training in dentistry involves supervision of dental students performing irreversible and invasive dental procedures on patients and this can be stressful for both the students as well as the faculty [18].

Pakistan is the fifth most populous country in the world and faces significant economic and socio-political challenges. Despite slow economic growth, there has been a significant increase in the number of private medical and dental institutions in the last two decades. Currently there are over 50 dental colleges offering undergraduate dental education programmes in Pakistan [19]. The results of the current study show that approximately 25% of participants showed features of moderate to severe depression on the PHQ-9 scale. The corresponding scores on DASS-21 identified 25% participants with moderate to severe depression; 24% participants with moderate to severe anxiety; and 17% participants with moderate to severe stress. Although there are no comparable studies on the frequency of mental health issues based on PHQ-9 and DASS-21 scales amongst dental faculty, these findings highlight that mental health issues amongst dental faculty are significant and warrant appropriate measures to support dental faculty experiencing depression, anxiety and stress.

Although the mental health of dental faculty has not been investigated specifically in Pakistan, a recent study on Pakistani dentists showed that work-related stress was lower among dentists over 40 years of age and those with a higher income threshold compared to junior dentists and those with a lower income respectively [20]. Previous studies on Pakistani dentists have also reported a strong correlation between workload and burnout and job dissatisfaction. Participants in the current study also reiterated the need to improve the financial package for the faculty to mirror the growing market inflation rates and improve job security. Although no significant effect of gender, experience, or specialty was identified in the current study, DASS-21 scores showed higher stress levels amongst participants less than 40-year age-groups. This may be related to the challenges of transition into professional life [21]. Young dentists in Pakistan lack appropriate guidance and resources to identify a suitable career pathway and may not have adequate financial resources to set up dental practice. Furthermore, social pressures to start a family, coupled with the financial responsibilities that come with it, can exacerbate their stress levels. It is pertinent to mention that a substantial proportion of dental faculty in both public and private sector dental institutions are engaged in private dental practice as a second source of earning after work at the dental institution. Although this was not explored in the current study, the pressures of doing a second job may lead to burnout and impact adversely on the mental health of dental faculty. This may be an interesting dimension to explore in future studies to gain a more holistic understanding of underlying factors affecting the mental health of dental faculty in Pakistan.

Previous studies have shown that doctors and dentists may experience higher levels of stress compared to the general population but in some cases, a teaching role for clinicians may actually reduce the stress [22]. A recent study on dental faculty at four dental schools in the United States showed high rates of burn out and loneliness amongst the participants [18]. Although there was no significant increase in burn out scores during the COVID-19 pandemic, the figures were higher compared to those reported amongst general public. On the other hand, a study involving 537 university teachers in Japan showed that approximately one third of the participants were at risk of mental illness due to work-related stress following the COVID-9 pandemic [23]. Two factors which contributed to poor mental health were difficulties in using IT for online classes and accessing IT support for their teaching assignments. Although the COVID-19 seems to have settled down, at least for now, the experiences of blended learning approaches have been adopted in dental education globally [24]. Therefore, there is a need to provide appropriate institutional support to the faculty for effective use of blended learning approaches in dental education.

Mental health of faculty and staff in dental education is a complex issue influenced by a multitude of factors, including high workloads, the pressure to excel in multiple domains, and the specific challenges inherent in dental practice and education. Participants in this study reported several factors which may impact adversely on the mental health of dental academics. Moderating workload for staff, promoting a healthy work life balance, appropriate financial remuneration and institutional support are essential to help faculty deliver dental education effectively. Addressing these issues is not only crucial for the well-being of the educators but is also essential for maintaining the quality of dental education and the overall health of the academic environment. As the body of research grows, it becomes increasingly clear that proactive measures and supportive policies are needed to safeguard the mental health of these valuable members of the academic community.

Mental health challenges amongst teaching faculty have prompted many academic institutions to implement measures to support the mental health of their faculty. These initiatives range from providing access to counseling and mental health services to introducing policies aimed at reducing work-related stress, such as workload management and flexible work arrangements (Guthrie et al., 2019). Some dental schools have also initiated wellness programs specifically tailored to address the unique needs of their faculty, incorporating strategies like peer support, mindfulness training, and stress management workshops (Morse et al., 2012). The institutional management of dental schools in Pakistan could also consider some of these measures to support the mental health of the faculty.

The main limitation of this study is that the data is reported from a single country with a unique socio-economic climate and findings may not be generalizable to other countries. Nevertheless, some of the challenges reported by the participants may well be applicable to dental faculty further afield and may become a barrier to their professional agency [25]. Dental academics represent the architects of dental education for future generations of dentists and their mental health and wellbeing is crucial to effective delivery of dental education. Future studies based on mixed methods in other settings are recommended to enhance the understanding regarding the scale of mental health issues amongst dental faculty so that remedial measures and support are provided early.

## Conclusion

This study provides useful insights into the frequency of mental health issues such as stress, anxiety, and depression among dental faculty at multiple institutions in Pakistan. The mean score on PHQ-9 was 6.51 (SD ± 5.4) while the mean DASS-21 score was 13.04 (SD ± 10.95). PHQ-9 Depression, and DASS-21 Depression, Anxiety, and Stress scores were all significantly positively correlated for the whole sample, and within each subgroup of each demographic factor. Job-related workload, lack of institutional support, financial limitations, and poor work life balance were identified as the main factors contributing adversely to the mental health of the participants. The study also identifies underlying factors which may compromise mental well-being of the faculty and provides recommendation to mitigate these challenges.

## Data Availability

The data underlying this article will be shared on reasonable request to the corresponding author.
